# Prevalence and Severity of Temporomandibular Disorders in Rheumatoid Arthritis Patients

**DOI:** 10.7759/cureus.21276

**Published:** 2022-01-15

**Authors:** Mohammad A Mustafa, Bader A AL-Attas, Fatma F Badr, Fatma M Jadu, Siraj O Wali, Yasser M Bawazir

**Affiliations:** 1 Rheumatology, University of Jeddah, Jeddah, SAU; 2 Emergency Department, King Fahad General Hospital, Jeddah, SAU; 3 King Abdulaziz University, Faculty of Dentistry, Jeddah, SAU; 4 Sleep Medicine and Research Center, King Abdulaziz University, Jeddah, SAU; 5 Rheumatology, King Abdulaziz University, Jeddah, SAU

**Keywords:** tempomandibular joint dysfunction., inflammatory arthritis, fonseca anamnestic index, rheumatoid arthritis, tempomandibular joint

## Abstract

Introduction

The temporomandibular joint (TMJ) is an important joint that plays major functions, including dental occlusion, mastication, and facial expressions. Different diseases can affect the TMJ, including chronic inflammatory arthritis. Rheumatoid arthritis (RA) is the most common inflammatory arthritis worldwide associated with TMJ dysfunction. In this study, we assess the prevalence of TMJ among RA patients based on the Fonseca Anamnestic Index.

Methods

Eighty-one patients with rheumatoid arthritis were interviewed by a trained physician to fulfill the Fonseca Anamnestic Index questionnaire. All participants underwent a medical file review to collect their sociodemographic data, RA duration, co-existing comorbidities, and different lab results.

Result

According to the Fonseca score, 29.6% had no temporomandibular disorder (TMD) among RA patients, while 39.5% had mild TMD. Only 6% had severe TMD. The female sex and increased body weight were associated with TMJ disease.

Conclusion

The majority of rheumatoid arthritis patients (70%) suffer from some degree of temporomandibular joint disorder.

## Introduction

The temporomandibular joint (TMJ) is a complex articular junction that connects the temporal bone and the mandible [1]. It plays a significant role in dental occlusion, facial expression, and mastication. Some multifactorial pathologies affect the TMJ, causing pain, swelling, clicking, crepitus, and functional loss [2,3]. Although genetics and environmental and aging processes are primary causes of temporomandibular disorder (TMD), inflammatory arthritis, such as rheumatoid arthritis (RA), juvenile idiopathic arthritis (JIA), and ankylosing spondylitis (AS) should also be considered [4]. In a systematic review, the prevalence of TMJ disorders varied widely from 6-93% of the general population [5]. This might be related to the lack of standardization and different evaluation tools that were used for the assessment.

Rheumatoid arthritis (RA) is a systemic autoimmune disease characterized by inflammatory arthritis along with extra-articular manifestations. It is the most common inflammatory arthritis, with a prevalence of 1% in the general population [6]. Young females are predominantly affected, with a usual age between 30-50 years [7]. However, RA can also occur in men, older age groups, and children, in which case it is known as juvenile rheumatoid arthritis (JIA) [8]. Commonly, it involves the small and large joints of the hands, including proximal interphalangeal joints (PIPs), metacarpophalangeal joints (MCPs), and wrists along with knees and metatarsophalangeal joints (MTPs). However, it has also been reported to a lesser extent in ankles, hips, elbows, shoulders, and temporomandibular joints (TMJs) [9].

The prevalence of TMD related to rheumatoid arthritis is underestimated, in large part due to a lack of a routine rheumatology exam for TMD in the rheumatology clinic [10]. Several assessment methods have been attempted in different studies to evaluate TMD. A systematic review showed that two-dimensional radiographs, cone-beam computed tomography (CBCT) and MRI, are the most commonly used in RA-TMJ assessment [11]. Sadura- Siecklucka et al. reported that half of RA patients suffer from TMJ problems based on clinical assessment by the visual analogue scale of pain and direct palpation of the TMJ [12]. Cordeiro et al. used the Research Diagnostic Criteria for Temporomandibular Disorders (RDC/TMD) questionnaire and CT scan for TMD assessment in RA patients. They found that three-quarters of the patients complained of orofacial pain, while CT scans detected TMJ radiographic changes in 90% of the study population [13]. Another questionnaire, the Fonseca questionnaire, was used solely to determine the link between RA and TMJ involvement; it was found that only 14.3% of RA patients did not have TMJ disease [14]. In Saudi Arabia, there are insufficient data about the prevalence of TMJ disorders associated with inflammatory arthritis. To our knowledge, there has been only one retrospective study of 123 JIA patients who reported TMD in 16% of RA patients [15].

This publication is the first to address the prevalence of TMJ involvement in RA patients in Saudi Arabia. This cross-sectional study aims to estimate the prevalence of TMJ involvement in RA patients based on the Fonseca questionnaire. In addition, the study was designed to identify the risk factors associated with TMD.

## Materials and methods

Design and setting

This cross-sectional study was conducted between June 2020 and December 2020 in the rheumatology clinic of King Abdulaziz University Hospital. A trained physician interviewed participants using the Fonseca et al. [16] questionnaire (Table 1). The questionnaire was translated from English to Arabic, then from Arabic back to English, and was then piloted using a focus group to ensure proper conversion to the native Arabic language. The Department of Biomedical Ethics Unit & Academic Affairs of King Abdulaziz University, Jeddah, approved this study (reference number 265-14). Consent was attained from all participants before they were interviewed to answer the questionnaire.

**Table 1:** The Fonseca Anamnestic Index questionnaire [16]. TMJ - temporomandibular joint

Question	Answer (yes, sometimes, no)
Is it hard for you to open your mouth?	
Is it hard for you to move your mandible from side to side?	
Do you get tired/muscular pain while chewing?	
Do you have a frequent headache?	
Do you have pain on the nape or stiff neck?	
Do you have earaches or pain in craniomandibular joints?	
Have you noticed any TMJ clicking while chewing or when you open your mouth?	
Do you clench or grind your teeth?	
Do you feel your teeth don’t articulate well?	
Do you consider yourself a tense (nervous) person?	

Inclusion and exclusion criteria

All rheumatoid arthritis patients aged 18 years or older were recruited. The exclusion criteria were: 1) all patients with rheumatological diseases other than RA, 2) patients with previous facial surgery, tumors, or trauma, 3) patients undergoing orthodontic treatment and having dental pain or ear pain, 4) patients with a history of recurrent seizures, 5) patients with migraine, 6) patients who had the parotid disease or sicca symptoms, 6) patients with trigeminal neuralgia and 7) patients on antiepileptic, antipsychotic or opioid medications.

Sampling and data collection

One hundred and fifty-six patients with RA were interviewed. However, 38 patients were excluded as per the exclusion criteria, and 37 patients refused to participate. Hence, only 81 patients compose our sample.

All patients were interviewed by a trained physician. The data collected included demographic data, duration of RA, history of chronic diseases, and the Fonseca Anamnestic Index (FAI).

RA patients who answered the questionnaire successfully underwent medical file review. The following data were collected: recent blood tests that included haemoglobin (Hb), vitamin D, calcium level, thyroid-stimulating hormone (TSH), erythrocyte sedimentation rate (ESR), C-reactive protein (CRP), rheumatoid factor (RF), anti-cyclic citrullinated peptide (anti-CCP) and serum creatinine.

This is a self-administered, low-cost, easily applied questionnaire for TMD assessment in the nonpatient population [16]. Thus, the FAI would serve as a preliminary TMD screening tool. After identifying the affected population, a complete clinical examination and further use of diagnostic instruments are required to confirm the diagnosis. The questionnaire also provides a severity index with less influence from the examiner and less variability in the measures.

Fonseca questionnaire follows the characteristics of a multidimensional evaluation. It is composed of 10 questions that screen for the presence of pain in the TMJ, head, and back; pain while chewing; parafunctional habits; movement limitations; joint clicking; perception of malocclusion; and sensation of emotional stress. Each question has three options: yes, no, or sometimes.

Statistical analysis

Data were entered, analyzed, and interpreted using SPSS version 25 (IBM Inc., Armonk, USA). Categorical variables were summarized as numbers and percentages. The Chi-square test was used to evaluate significance. However, continuous variables were summarized as means and standard deviations for normally distributed variables and as medians and Q1-Q3 for non-normally distributed variables. The Mann-Whitney U-test was used as a test of significance for non-normally distributed variables. Spearman correlation was used to correlate non-normally distributed data with each other or with normally distributed variables. Relevant pie charts and bar charts were used to visualize the data. P-value <0.05 was considered statistically significant.

## Results

Out of 81 RA patients, 95% were females, 68% were Saudi, and 97% were non-smokers. The mean age was 47 years. The median body weight was 77.5 kg with Q1 to Q3 of 65 to 90 kg, while the median height was 156 cm with Q1 to Q3 of 151 to 160 cm. BMI was 30.5 kg/m^2^ with Q1 to Q3 of 26.6 to 36.9 kg/m^2^. The median duration of RA was 60 months, with Q1 to Q3 of 24 to 132 months. Twenty-one percent of patients had diabetes mellitus (DM), 18.5% had hypertension, and 5% had ischaemic heart disease (IHD). Furthermore, 3.7% of patients had a stroke (Table 2).

**Table 2:** Demographic, anthropometric, and medical characteristics of the rheumatoid arthritis patients (n=81) DM - diabetes mellitus; HTN - hypertension; IHD - ischemic heart disease

Demographic characteristics			
		N	%
Sex	Female	77	0.951
	Male	4	0.049
Nationality	Non-Saudi	26	0.321
	Saudi	55	0.679
Smoker	No	79	0.975
	Yes	2	0.025
		Mean	Standard deviation
Age in years		47	13
Anthropometric measurements			
		Median	Q1-Q3
Weight		77.5	65-90
Height		156	151-160
BMI		30.5	26.6-36.9
Medical history			
		Median	Q1-Q3
Duration of rheumatoid arthritis in months		60	24-132
		N	%
DM	No	64	0.79
	Yes	17	0.21
HTN	No	66	0.815
	Yes	15	0.185
Stroke	No	78	0.963
	Yes	3	0.037
IHD	No	77	0.951
	Yes	4	0.049

Thirty-one patients (38.3%) were rheumatoid factor (RF)-seropositive. However, only 13 (16%) patients were anti-cyclic citrullinated peptide (anti-CCP)-seropositive; 44.4% of patients were seropositive either RF-seropositive or anti-CCP seropositive. The median RF was 12 with Q3 of 52. In addition, the median anti-CCP was 23, with Q3 of 100.

**Figure 1:**
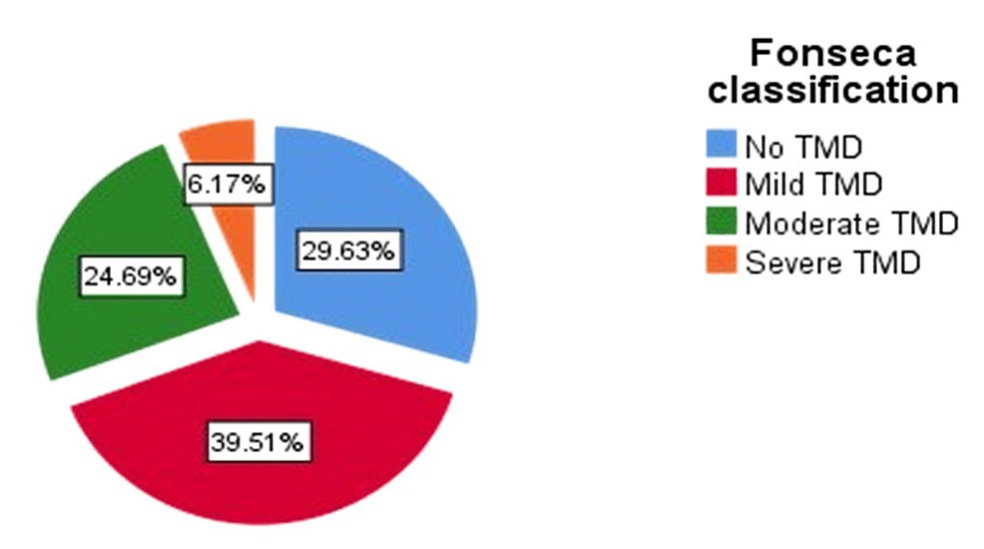
Prevalence of TMD among RA patients (n=81) according to Fonseca score TMD - temporomandibular disorder; RA - rheumatoid arthritis

Regarding the prevalence of TMD among RA patients, according to the Fonseca score, 29.6% had no TMD, while 39.5% had mild TMD. Only 6% had severe TMD (Figure 1).

When comparing RA patients with TMD and those with no TMD regarding the different variables shown in Tables 3 and 4, there were statistically significant differences only according to sex and hemoglobin A1C (HA1C; p=0.042 and 0.009, respectively). However, nationality, smoking, DM, HTN, IHD, and stroke were non-significant. Moreover, age, weight, height, BMI, RA duration, disease activity score (DAS), hemoglobin, fasting blood glucose (FBS), urea, creatinine, calcium, vitamin D, thyroid-stimulating hormone (TSH), C-reactive protein (CRP), erythrocyte sedimentation rate (ESR), RF, and anti-CCP were non-significant (Table 4). Sixty-nine percent of seropositive patients vs. 71% of seronegative patients had TMD; however, this percentage was non-significant (p=0.870; see Table 3).

**Table 3:** Comparison between RA patients with TMD and those with no TMD regarding categorical variables RA - rheumatoid arthritis; TMD - temporomandibular disorder; X^2 ^- Chi-square test; DM - diabetes mellitus; HTN - hypertension; IHD - ischemic heart disease; RF - rheumatoid factor; anti-CCP - anti-cyclic citrullinated peptide

		TMD (N=57)		No TMD (N=24)		Χ^2^	p-value
		N	%	N	%		
Sex	Female	56	72.7%	21	27.3%	4.154	0.042*
	Male	1	25.0%	3	75.0%		
Nationality	Non-Saudi	16	61.5%	10	38.5%	1.432	0.231
	Saudi	41	74.5%	14	25.5%		
Smoker	No	55	69.6%	24	30.4%	0.863	0.353
	Yes	2	100.0%	0	0.0%		
DM	No	47	73.4%	17	26.6%	1.376	0.241
	Yes	10	58.8%	7	41.2%		
HTN	No	45	68.2%	21	31.8%	0.819	0.366
	Yes	12	80.0%	3	20.0%		
Stroke	No	56	71.8%	22	28.2%	2.050	0.152
	Yes	1	33.3%	2	66.7%		
IHD	No	54	70.1%	23	29.9%	0.043	0.835
	Yes	3	75.0%	1	25.0%		
RF- or anti-CCP-seropositive	No	32	71.1%	13	28.9%	0.027	0.870
	Yes	25	69.4%	11	30.6%		

**Table 4:** Comparison between RA patients with TMD and those with no TMD regarding quantitative variables RA - rheumatoid arthritis; TMD - temporomandibular disorders; BMI - body mass index; DAS - disease activity score; CBC - complete blood count; FBS - fasting blood glucose; HA1C - hemoglobin A1C (glycated hemoglobin); TSH - thyroid-stimulating hormone; CRP - C-reactive protein; ESR - erythrocyte sedimentation rate; RF - rheumatoid factor; anti-CCP - anti-cyclic citrullinated peptide The Mann-Whitney U test was used.

	TMD		No TMD		
	Mean	Standard deviation	Mean	Standard deviation	p-value
Age	45	12	52	14	0.062
Weight	82.2	24.3	74.8	22.3	0.109
Height	156.3	8.9	154.2	13.9	0.427
BMI	34.2	11.9	32.2	12.4	0.271
Duration of rheumatoid arthritis in months	95.1	82.3	69.8	67.7	0.208
DAS 28 score	3.3	.9	3.4	1.2	0.431
CBC	11.9	1.6	12.1	1.7	0.780
FBS	7.4	10.1	6.4	1.8	0.079
HA1C	5.7	.7	6.3	1.1	0.009*
Urea	4.4	5.3	5.3	6.9	0.634
Creatinine	55.3	19.3	62.3	11.8	0.222
Calcium	2.2	.1	2.3	.1	0.103
Vitamin D	54.2	31.3	52.0	20.7	0.808
TSH	2.5	2.2	4.2	9.2	0.792
CRP	11.1	9.4	10.1	8.6	0.877
ESR	32.9	19.7	36.9	19.5	0.211
RF	69.4	120.3	104.9	219.9	0.959
Anti-CCP	139.7	265.6	103.4	212.3	0.979

The prevalence of TMD was higher among females than males (72.7% vs. 25%, p=0.042; see Table 3). The mean HA1C was higher among those with no TMD than among those with TMD (6.3 vs. 5.7, p=0.009; see Table 4). Furthermore, there was a statistically significant weak negative correlation between the total Fonseca score and FBS and HA1C (SP=-0.220, p=0.048, and SP=-0.222, p=0.046, respectively; see Table 5).

**Table 5:** Spearman correlation between the total Fonseca score and anthropometric measurements and RA history of patients RA - rheumatoid arthritis; BMI - body mass index; DAS: - disease activity score; CBC - complete blood count; FBS - fasting blood glucose; HA1C - hemoglobin A1C (glycated hemoglobin); TSH - thyroid-stimulating hormone; CRP - C-reactive protein; ESR - erythrocyte sedimentation rate; RF - rheumatoid factor; Aanti-CCP - anti-cyclic citrullinated peptide

	Total Fonseca score	
	Correlation coefficient	Significance
Anthropometric measurements		
Weight	0.235	0.034*
Height	0.114	0.309
BMI	0.188	0.093
Age	-0.106	0.348
RA history of patients		
DAS28 score	-0.107	.0344
Duration of rheumatoid arthritis	-0.079	0.483
CBC	0.049	0.661
FBS	-0.220	0.048*
HA1C	-0.222	0.046*
Urea	-0.068	0.544
Creatinine	-0.064	0.570
Calcium	-0.087	0.437
Vitamin D	-0.057	0.613
TSH	0.006	0.957
CRP	-0.001	0.990
ESR	-0.092	0.414
RF	0.017	0.879
Anti-CCP	0.037	0.745
Body weight	0.239	0.032*

**Figure 2:**
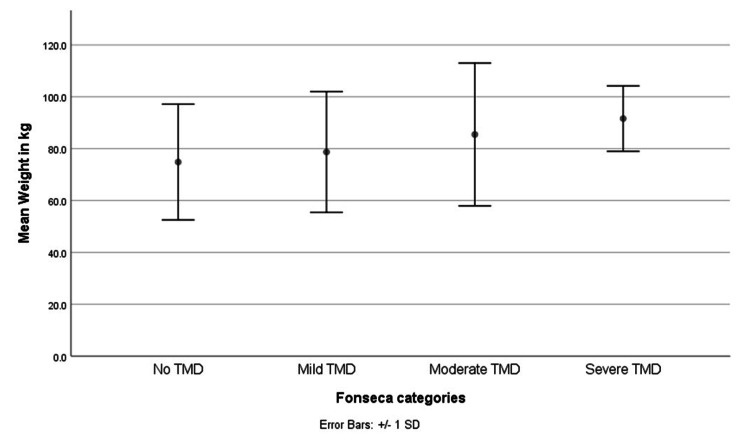
Comparison of the Fonseca classification categories according to patient body weight TMD - temopormandibular disorder

When correlating the total Fonseca score to different quantitative variables, age, height, BMI, RA duration, DAS score, hemoglobin, urea, creatinine, calcium, vitamin D, TSH, CRP, ESR, RF, and anti-CCP showed non-significant correlation. However, there was a statistically significant weak positive correlation between the total Fonseca score and weight (SP=0.235, p=0.034; see Table 5). Furthermore, when correlating the Fonseca classification categories to the body weight of patients, there was also a weak positive statistically significant correlation (SP=0.239, p=0.032). However, although the mean body weight increased while increasing the severity of TMD (Figure 2), this was statistically non-significant using one-way ANOVA to compare the means (p=0.330).

Reporting

The Strobe guidelines for observational studies were used to conduct and report this manuscript [17].

## Discussion

The Fonseca questionnaire is a previously validated, affordable, and efficient method for assessing TMD prevalence in RA patients [16,18]. It generates a score that classifies patients into one of four groups: no TMD, mild TMD, moderate TMD, and severe TMD [16]. This study revealed that 70% of RA patients suffer from TMD to varying degrees. This is more than double the global prevalence of TMD in the community [19]. The majority of our sample suffered from mild TMD (39.5%), followed by moderate TMD (24.7%) and severe TMD (6.2%). Similarly, a nationwide study in Taiwan revealed that RA patients are 2.5 times more likely to develop TMD than non-RA patients [20]. Additionally, Yamakawa et al. found that the prevalence of TMD in RA patients was 67.6% [21]. Other authors found a lower prevalence of TMD in RA patients (56.7%). However, the sample size was smaller than the sample in the current study [12].

This study found that female sex was associated with a greater risk of TMD, consistent with the literature [20]. This is scientifically plausible since females are more susceptible to developing both rheumatoid arthritis and TMD [6,18]. In addition, there was a significant positive correlation between body weight and the total Fonseca score. However, although the mean body weight increased with increasing severity of TMD, this increase was statistically non-significant by one-way ANOVA. This may be due to the small number of patients remaining in each group after splitting them into the four Fonseca categories. Thus, the risk of TMD seems to increase incrementally as body weight increases. This is interesting because it suggests that there is no weight threshold above which the risk of developing TMD increases. TMD could be added to the list of chronic illnesses that are associated with increased body weight. We found a significant negative association between the total Fonesca score and two blood compounds: hemoglobin A1C (HA1C) and fasting blood sugar (FBS). Similarly, in a previous research paper, reactive hypoglycemia was associated with temporomandibular joint pain [22]. The reason behind this association is unclear. Future studies with larger samples may refute this finding, especially because the association is weak. Furthermore, our study did not find a significant association between age and TMD. However, this may be partly because patients younger than 18 years old were not included.

Being RF- or anti-CCP-seropositive does not seem to be associated with TMD. However, the prevalence of TMD was slightly higher among seronegative patients than among seropositive patients (71% vs. 69%), which is consistent with a previous study that found lower TMJ pain among seropositive patients than among seronegative patients. This previous study suggested that the TMJ pressure pain threshold was modulated by systemic mediators rather than local inflammatory mediators [23]. The duration of RA seems to have an inconsistent influence on the risk of TMD. Although it was reported to correlate with TMD positively [21], our study found that TMD was not significantly associated with the duration of RA. This is slightly perplexing since RA is a progressive condition. Longitudinal studies may be helpful in elucidating the effect of RA duration on TMD development and progression.

This study has several limitations. Our sample did not have an equal sex distribution due to the much higher prevalence of RA among females. Although questionnaire-related bias is a valid limitation, we tried to reduce it by training the interviewer to conduct the survey in a consistent manner. The questionnaire was also translated twice and piloted prior to data collection. Ground truth using biopsy or radiographic assessment of TMD was not performed; however, the questionnaire used was globally validated and did not involve the risks of surgery or ionizing radiation. In addition, this study is limited by the inability to determine the temporal link between TMD and RA, similar to all observational studies, but this was not the purpose of this study. Future prospective studies that also assess oropharyngeal dysphagia [24], which is a serious consequence of TMD in RA patients, are recommended.

## Conclusions

The temporomandibular joint disorders among RA patients are common that require further attention by treating physicians. This study showed that most rheumatoid arthritis patients (70%) suffer from some degree of temporomandibular joint disorder. Although this study failed to correlate the duration of RA and TMDs, female sex and increased body weight were identified risk factors for TMDs in RA patients.
